# Identification of Serum miRNA-423-5p Expression Signature in Somatotroph Adenomas

**DOI:** 10.1155/2019/8516858

**Published:** 2019-07-17

**Authors:** Sida Zhao, Jianhua Li, Jie Feng, Zhenye Li, Qian Liu, Peng Lv, Fei Wang, Hua Gao, Yazhuo Zhang

**Affiliations:** ^1^Beijing Neurosurgical Institute, Capital Medical University, Beijing, China; ^2^Department of Neurosurgery, Binzhou People's Hospital, Binzhou, Shandong, China; ^3^Chinese Medical Association, Beijing 100710, China; ^4^Department of Neurosurgery, Provincial Hospital Affiliated to Anhui Medical University, China; ^5^Department of Neurosurgery, Beijing Tiantan Hospital, Capital Medical University, Beijing, China; ^6^Beijing Institute for Brain Disorders Brain Tumor Center, Capital Medical University, Beijing, China; ^7^China National Clinical Research Center for Neurological Diseases, Beijing, China; ^8^Key Laboratory of Central Nervous System Injury Research, Beijing, China

## Abstract

Circulating miRNAs are novel disease biomarkers that are valuable for diagnosis and prognosis. But the circulating miRNAs profile in somatotroph adenomas is still unknown. Therefore, serum exosomal miRNAs expression profiling in somatotroph adenomas was performed on 6 somatotroph adenomas and 6 normal controls. From the exosomal miRNAs expression profiling, we found 169 miRNAs differently expressed between somatotroph adenomas and healthy pituitary samples (p< 0.05, FC > 2). Among the 169 miRNAs, miR-423-5p was expressed lower in somatotroph adenomas than in healthy pituitary samples, which was proved by miRSCan Panel Chip™ qPCR. PTTG1 and SYT1 were the target mRNAs of miR-423-5p, and transcriptomics and proteomics profile both indicated the high expression of PTTG1 and SYT1 in somatotroph adenomas. H-scores were 223.1 ± 34.7 for PTTG1 and 163.4 ± 42.3 for SYT1 in 62 somatotroph adenomas specimens and 84.2 ± 21.3 for PTTG1 and 47.4 ± 17.2 for SYT1 in 6 healthy pituitary specimens by IHC. miR-423-5p inhibited the expression of SYT1 and PTTG1 at the mRNA and protein levels. Dual luciferase reporter gene assay shown was significantly reduced in the presence of miR-423-5p in GH3 cells transfected with wild-type PTTG1 3'UTR luciferase reporter plasmid but not reduced when transfected with the mutation PTTG1 3'UTR luciferase reporter plasmid (p<0.01).* In vitro* experiments showed that miR-423-5p induced cell apoptosis, inhibited cell proliferation, and reduced growth hormone release and migration of GH3 cells. The activity of miR-423-5p in GH3 cell was nearly blocked by its inhibitor. These results verified the central role of low miR-423-5p in promoting tumorigenesis in somatotroph adenomas. PTTG1 may act as biomarkers for clinical treatment of somatotroph adenomas.

## 1. Introduction

Somatotroph adenomas were the second most common functional pituitary adenomas and usually occur with cardiovascular, respiratory, and cerebrovascular disease [1], accompanied by a higher risk of mortality compared to people without the disease [2]. High secretion of growth hormone is often the reason of acromegaly in adults. Among surgery, medical treatment (somatostatin agonists) and radiotherapy treatment, surgery is the first choice [3]. Despite the surgery, the remission rate is approximately 50% and medical treatment is recommended. Based on immunohistochemistry (IHC) and molecular biology analysis, patients are divided into groups for personalized medicine [4]. In clinics, surgery cannot be performed for some patients; thus no tumor samples are available for the analysis of biomarkers such as somatostatin receptor type 2, sst5r, aryl hydrocarbon receptor-interacting protein (AIP), and granulation pattern. Advances in liquid biopsy allow testing of many circulating biomarkers for progression and treatment of diseases including hepatocellular carcinoma [5] and lung cancer [6].

Exosomes are a group of 30 to 100 nm nanoscale membrane vesicles derived from multivesicular endosomes by cell endocytosis [7]. Micro-RNAs (miRNAs) are a group of small single-stranded noncoding RNAs with lengths of 18-25 nucleotides [8]. MiRNAs can be secreted from cells into body fluids such as blood, urine, breast milk, and saliva via exosomes [9]. They bind to the target mRNAs and negatively regulate gene expression after transcription [10, 11]. Studies showed that exosome-derived miRNAs are related to tumor development and progression by affecting biological pathways and pathological states [12]. Significant difference of exosome miRNAs existed in many different types of tumors, which provided a chance for exploring serum-derived exosomes as new tumor biomarkers [13]. MiRNA-34a is a negative factor for AIP in somatotroph adenomas with an invasive phenotype and resistance to somatostatin analogs [14]. MiRNA-93 significantly inhibits the sensitivity of MMQ cells to treatment with dopamine agonists [15] (DAs). MiRNA-137 is important by affecting WIF1 promoter methylation in nonfunctioning pituitary adenomas [16]. Altered miRNA profile target genes in somatotroph adenomas such as high mobility group A1 (HMGA1), HMGA2, and E2F are playing important role in pituitary tumorigenesis [17].

In this study, we investigated the difference of serum exosomal-derived miRNA expression signature between somatotroph adenomas and healthy pituitary specimens to explore the specific miRNA biomarkers in somatotroph adenomas. Gene targets from miRNA profiling were filtered by miRanda and Targetscan and compared with the transcriptomics and proteomics profiling data of somatotroph adenoma tissues. Furthermore, we explored the potential functions of the candidate miRNA on cell proliferation, apoptosis, migration and invasion, and growth hormone release in GH3 cells.

## 2. Materials and Methods

### 2.1. Patients and Tissue Specimens

Somatotroph adenomas were obtained from patient of Beijing Tiantan Hospital. Fresh tumor tissue samples from these patients were frozen at -80°C in isopentane and stored in liquid nitrogen. Pituitary adenoma samples were analyzed by immunohistochemistry and transmission electron microscopy. Diagnoses of somatotroph adenoma were based on pathological and electron microscopic examination as previously described [18]. Healthy pituitary samples were acquired from postmortem tissues dying from accidents.

This study was approved by the ethics committees of Beijing Tiantan Hospital (KY2013-015-02). Informed consent was obtained from all enrolled subjects, and the study was performed in full compliance with all principles of the Declaration of Helsinki.

#### 2.1.1. Exosomal RNA Extraction and Sequencing

Serum samples from 6 somatotroph adenomas and 6 healthy pituitary samples were used for exosomal RNA sequencing. PureExo Exosome Isolation Kits (101Bio, Palo Alto, CA) were used to isolate serum exosomes. Exosomes appeared as a separate fluffy layer after vortexing serum samples with solutions provided by kits and microcentrifugation. 3*μ*g RNA of each sample was used as input material for RNA sample preparations. First, ribosomal RNA was removed by Epicentre Ribo-zeroTM rRNA Removal Kit (RZH1046, Epicentre). Subsequently, sequencing libraries were generated using the rRNA-depleted RNA by NEBNext® UltraTM Directional RNA Library Prep Kit for Illumina® (E7420L, NEB) following manufacturer's protocol. First strand cDNA was synthesized using random hexamer primer. Second, strand cDNA synthesis and making, which incorporates dUTP into the second strand, converts the cDNA. Double-stranded DNA was repaired via exonuclease/polymerase activities and then adenylation to the 3' end was add. After adapter ligation and library amplification, the library fragments were purified with AMPure XP system (Beckman Coulter, Beverly, USA) in order to select fragments of preferentially 150~200 bp in length. The strand marked with dUTP is not amplified, allowing strand-specific sequencing. At last, products were purified (AMPure XP system) and library quality was assessed on the Agilent Bioanalyzer 2100 system. After cluster generation, the libraries were sequenced on an Illumina Hiseq X platform and 150bp paired-end reads were generated.

#### 2.1.2. miRSCan Panel ChipTM qPCR

The samples used for exosome RNA sequencing were also used for miRSCan Panel ChipTM qPCR. On the panels, there were 172 biomarkers including 163 miRNA biomarkers related to tumorigenesis, 3 spike-in controls, 4 hemolysis controls, and 2 potential endogenous controls. One *μ*L 0.2–0.4 ng RNA sample was assayed by Bioanalyzer® smRNA kits. MiRNAs were polyadenylated and converted into cDNA by QB-universal primer reverse transcription in a single step. QPCR reaction mixtures of cDNA and SYBR® Green master mix were loaded onto miRSCan Pan Cancer Chip 1 & 2 (Panel Chip™). Reactions were carried out using PanelStation™. Presence of hemolysis was determined by evaluating the number of blood cells with miRNAs in the sample. RT spike-in RNA (QB-spikein-1) was used as a control for reverse transcription. QPCR spike-in DNA (QB-spikein-2) was used as a control for qPCR. The cutoff point of significance was p-value ≤ 0.05 and fold-change ≥ 2.

#### 2.1.3. Microarray Hybridization and NanoLC-MS/MS Analysis and qRT-PCR

Microarray hybridization, nanoLC-MS/MS analysis and qRT-PCR was done with reference to our previous articles [19]. Each slide was hybridized with 1.65*μ*g Cy3-labeled cRNA using a Gene Expression Hybridization Kit (Agilent Technologies) and a hybridization oven (Agilent Technologies). After 17 hours of hybridization, the slides were washed with Gene Expression Wash Buffer Kit (Agilent Technologies) and scanned on Microarray Scanner (Agilent Technologies). RT-qPCR was operated by using the Applied Bio-systems 7500 Fast System (Life Technologies). The fold-change in differential expression for each gene was calculated using the comparative CT method (also known as the 2^−∆∆CT^ method).

The samples separated via capillary high-performance liquid chromatography were subsequently analyzed using a Triple TOF 5600þ system (AbSciex, USA). Protein identification and proteome annotation were performed using the ProteinPilotTM software package 4.5 (Applied Biosystems) and searched against the SwissProt database (March 2013) using the Mascot 2.2 search engine (Matrix Science, London, UK).

#### 2.1.4. Immunohistochemistry

62 somatotroph adenomas specimens and 6 healthy pituitary specimens were used for (immunohistochemistry). Formalin fixation and paraffin embedding of tissues were performed overnight at 4 C. The samples were subsequently analyzed via immunohistochemistry (IHC) using a routine IHC method (Hong et al., 2014). IHC was performed using specific antibodies against SYT1, PTTG1. The following four antibodies were added to the tissue samples for overnight incubation at 4°C, antibodies anti-SYT1 (1:500, ab133856, Abcam, Cambridge, UK) and anti-PTTG1 (1:1000, ab199239, Abcam, Cambridge, UK). Staining intensity was stratified on a scale of 0–3 (0 = no staining, 1 = weak, 2 = moderate, and 3 = strong staining [H-score = scale × (percentage of strong staining); 1.0 (% weak), 2.0 (% moderate), 3.0 (% strong)] to give a score ranging from 0 to 300.

#### 2.1.5. MTS Assay

GH3 cells were plated into 96-well dishes with 10,000 cells and 100 *μ*L medium per well and incubated for 24h, 48h, and 72h. Indicated concentrations (100 nM) of rat-miR-423-5p, negative control mimics (miR-423-5p-NC), and inhibitor (RIBOBIO, Guangzhou, China) were transfected into each well using Opti-MEM (Gibco, Carlsbad, CA, USA) and Lipofectamine 3000 (Invitrogen) according to the manufacturer's instructions.

#### 2.1.6. Cell Migration Experiment

Cell migration experiment was measured by using transwell assay. The protocol was from our previous articles [16]. Cell migration was measured using fibronectin- and Matrigel-coated polycarbonate filters, respectively, and modified transwell chambers (Corning). GH3 cells (5×10^4^ cells) were added into the upper chambers. Migrating cells that adhered to the lower membrane were fixed in 4% paraformaldehyde and stained using hematoxylin (Zhongshan Company). Experiments were performed in triplicate time.

#### 2.1.7. Elisa Assay

Growth hormone level was detected by Elisa kit (APPLYGEN) according to the protocol. The absorbance at 450 nm of each well was measured using an ELISA plate reader (Thermo Fisher).

#### 2.1.8. Flow Cytometry

The apoptosis of GH3 cell was detected by using Annexin V-FITC/PI kit (BD Biosciences, US). Cells were acquired and stained following the instruction of the manufacturer. Cells were analyzed using flow cytometry (ImageStream mk II, USA). Data was analyzed by using IDEAS Application v6.1 software.

#### 2.1.9. Western Blot Analysis

Protein extraction from GH-PAs and healthy pituitary gland tissues was performed using a total protein extraction kit (Cat. # 2140; Millipore, Billerica, MA, USA). Protein concentrations were measured using the BCA protein assay kit (23225, Pierce, Rockford, IL, USA). Soluble proteins (30 *μ*g) were separated by electrophoresis in 10% sodium dodecyl sulfate polyacrylamide gels, transferred to nitro-cellulose membranes, and incubated with blocking buffer (5% nonfat milk) in Tris-buffered saline/Tween-20 (TBST) for 1 h at room temperature. Membranes were then probed overnight with the corresponding primary antibody at 4°C followed by three 10-min washes with TBST. Subsequently, membranes were incubated with secondary antibodies conjugated to horseradish peroxidase at room temperature for 1 h followed by three 10-min washes with TBST. Blots were visualized by enhanced chemiluminescence, and densitometry was performed on an Amersham Imager 600 (GE). Anti-PTTG1 antibody (ab199239, dilution factor 1:3000), anti-SYT1 (ab133856, dilution factor 1:2000), and anti-GAPDH antibody (Cat. # G1020V, dilution factor 1:8000) were used for Western blot analysis. The final data were subjected to grayscale scanning and semiquantitative analysis using ImageJ software (https://imagej.nih.gov/ij/download.html). The final data were subjected to grayscale scanning and semiquantitative analysis using ImageJ software (https://imagej.nih.gov/ij/download.html).

#### 2.1.10. Dual Luciferase Reporter Gene Assay

miRTarBase (http://mirtarbase.mbc.nctu.edu.tw/php/index.php) was used to predict the target mRNAs of miRNA. Sequences of SYT1 and PTTG1 were acquired from Ensembl (http://asia.ensembl.org/index.html). Interacting sequences between miRNAs and mRNAs were analyzed by Global Align in Blast (https://blast.ncbi.nlm.nih.gov).

The PTTG1 sequence that incorporated binding sites with miR-423-5p was amplified through performing PCR at first and were then inserted into pmirGLO (Promega, Madison, WI, USA) in order to establish the reporter vector of wild-type pmirGLO-PTTG1. For another, the reporter vectors of mutation pmirGLO-PTTG1 were constructed through mutating the binding sites of PTTG1 to miR-423-5p. With the help of Lipofectamine 3000 transfection kit (Promega, Madison, WI, USA), pmirGLO-XIST Mut, pmirGLO-XIST Wt, pmirGLO-ROR1 Mut, and pmirGLO-ROR1 Wt were, respectively, cotransfected with miR-423-5p mimic or miR-423-5p-NC into GH3 cells. After 48 h transfection of wild-type or mutation of PTTG1 cotransfected with miR-423-5p, the luciferase reporter gene activity of cells was determined following the guidance of dual luciferase detection kit[20] (Promega, Madison, WI, USA).

#### 2.1.11. Bioinformatics Analysis and Statistical

Kyoto Encyclopedia of Genes and Genomes (KEGG) were used to analyze the pathway enrichment of the molecules in somatotroph adenomas. Unpaired Student's* t*-tests and chi-square (Fisher's exact) tests were used to compare quantitative and qualitative data.

All statistical analyses were conducted using SPSS Statistics Version 22 (IBM Corporation, Armonk, New York, USA). An unpaired Student's t test and a chi-square (Fisher's exact) test were used to compare quantitative and qualitative data. P value of less than 0.05 was considered significant.

## 3. Result

### 3.1. Expression Profiling of Somatotroph Adenoma Exosome miRNAs

**Figure 1:**
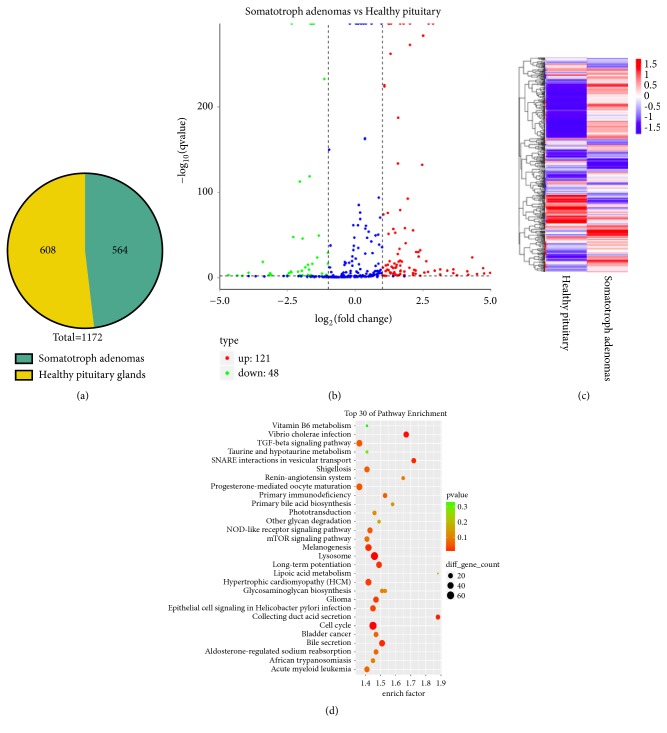
*MiRNA expression profiling of somatotroph adenomas and healthy pituitary samples. *(a): Exosome sequence profiling found 564 miRNAs in somatotroph adenomas and 608 healthy pituitary samples. (b) The volcano plot showed differently expressed miRNAs between somatotroph adenomas and healthy pituitary samples, red point indicated the 121 upregulated miRNAs, and green point indicated the 48 downregulated miRNAs in somatotroph adenomas. Blue point represented the miRNAs that were no difference between somatotroph adenomas and healthy pituitary samples. (c) Cluster analysis of differently expressed miRNAs between somatotroph adenomas and healthy pituitary samples. (d) 511 target mRNAs of hsa-miR-320a and hsa-miR-423-5p were enriched in different signal pathways by KEGG.

Exosomes were derived from the sera of six somatotroph adenomas and six healthy pituitary samples. Serum-derived exosome sequencing profiling identified 571 miRNAs in somatotroph adenomas including 564 known and 7 novel miRNAs. And in healthy pituitary samples, there were 608 known miRNAs and 6 novel miRNAs. There were 169 known miRNAs differently expressed between somatotroph adenomas and healthy pituitary samples, including 121 upregulated miRNAs and 48 downregulated miRNAs in somatotroph adenomas samples (p < 0.05, fc > 2, Supplementary Table 1). The 169 miRNAs can be mapped to 6377 mRNAs (Supplementary Table 2). The result of pathway enrichment indicated 6377 mRNAs were mainly enriched in Lysosome pathway, Cell cycle pathway, and Vibrio cholerae infection pathway by KEGG (Figure 1).

### 3.2. Validating Differently Expressed miRNAs by miRScanTM Panel Chip

miRScanTM Panel Chip qPCR was used to validate the expression of exosomal miRNAs in somatotroph adenomas and healthy pituitary samples by exosomal sequencing data. The result showed 16 miRNAs were differently expressed with significance on the panel between somatotroph adenomas and healthy pituitary glands (p < 0.05, fc ≥ 2). Of the 16 miRNAs, 8 were also significantly expressed in sequencing data: including hsa-miR-199a-5p, hsa-miR-30b-5p, hsa-miR-23a-3p, hsa-miR-223-3p, hsa-miR-103a-3p, hsa-miR-221-3p, hsa-miR-320a, and hsa-miR-423-5p. Hsa-miR-320a and hsa-miR-423-5p were the only two miRNAs with the same tendency in both exosome miRNA sequencing and miRSCan Panel ChipTM qPCR result in somatotroph adenomas. Hsa-miR-320a and hsa-miR-423-5p both were proved to be lower expressed by exosome miRNA sequencing and miRSCan Panel ChipTM qPCR result in somatotroph adenomas. None of other miRNAs were both lower and higher expressed in exosome miRNA sequencing and miRSCan Panel ChipTM qPCR result in somatotroph adenomas. Fold-change values of the 8 miRNAs were shown in Table 1. The target mRNAs of hsa-miR-320a and hsa-miR-423-5p were totally 511, with 255 target mRNAs of hsa-miR-320a and 256 target mRNAs of hsa-miR-423-5p (Supplementary Table 3).

### 3.3. Exosomal Expression Profiling Analysis Combined with Transcript and Proteomics Profiles

**Figure 2:**
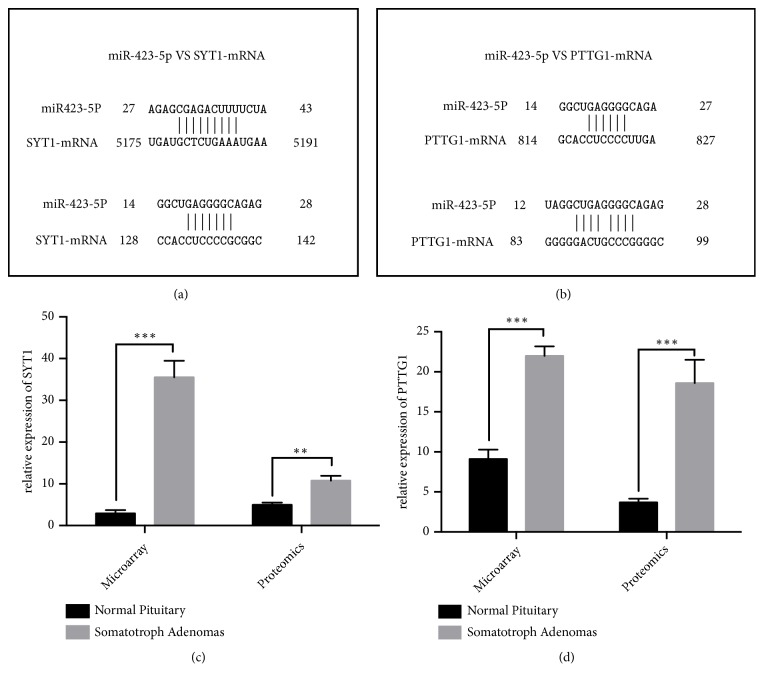
*Predicting and validating the expression of miR-423-5ptarget molecules.* (a, b): MiR-423-5pinteracting sequences with SYT1 and PTTG1. (c, d): Expression of SYT1 and PTTG1 at the mRNA and protein levels.

Microarray and proteomic analysis of somatotroph adenomas and healthy pituitary samples were used to test the expression of target mRNAs and proteins of hsa-miR-320a and hsa-miR-423-5p. The results of microarray and proteomic data were listed in Supplementary Tables 4 and 5, respectively. Of the 511 predicted target mRNAs, 106 were also significantly expressed in microarrays data of somatotroph adenomas (p < 0.05, fold-change >2). Eight mRNAs were mapped to corresponding proteins in proteomics data of somatotroph adenomas (p < 0.05, fold-change >1.5). Integrating the transcriptomics and proteomics profiling of somatotroph adenomas, we revealed PTTG1 and SYT1 were differently expressed at the protein and mRNA levels between somatotroph adenomas and healthy pituitary samples; these two genes were the targets of miRNA-423-5p. The mRNA sequences of PTTG1, SYT1, and miR-423-5p were analyzed and the result showed a series of complementary sequences between PTTG1 and miR-423-5p. Part of sequences of SYT1 were also complementary with part of the sequence of miR-423-5p (Figures 2(a) and 2(b)).

Microarray analysis showed that mRNA levels were 12.17-fold higher for SYT1 and 2.41-fold higher for PTTG1 in somatotroph adenomas than in healthy pituitary glands. Proteomics data indicated the protein levels were 2.16-fold higher for SYT1 and 4.96-fold higher for PTTG1 in somatotroph adenomas than in healthy pituitary glands (Figures 2(c) and 2(d)).

### 3.4. PTTG1 and SYT1 Expression Profiles Correlated with Clinicopathologic Parameters in 62 Somatotroph Adenomas

**Figure 3:**
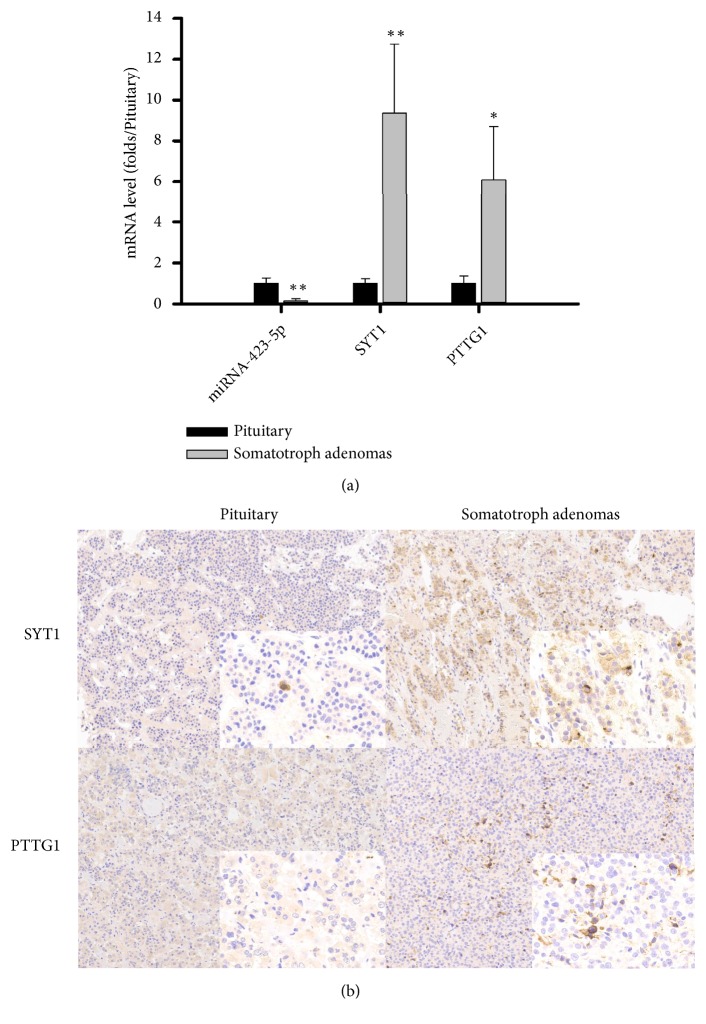
*Levels of miR-423-5ptarget genes PTTG1 and SYT1 in pituitary and somatotroph adenomas.* (a): MRNA levels for PTTG1 and SYT1 in pituitary and somatotroph adenomas. ^*∗*^p < 0.05, ^*∗∗*^p < 0.01 (b): IHC of PTTG1 and SYT1.

RT-PCR result showed the mRNA levels of PTTG1 and SYT1 in somatotroph adenomas samples were higher than those in healthy pituitary glands. Fold-change was 9.37 ± 3.37 for PTTG1 and 6.08 ± 2.62 for SYT1 (Figure 3(a)). IHC was used to analyze the H-scores of PTTG1 and SYT1 for 62 somatotroph adenomas and 6 healthy pituitary samples (Figure 3(b)). H-Scores were 223.1 ± 34.7 for PTTG1 and 163.4 ± 42.3 for SYT1 in 62 somatotroph adenomas specimens and 84.2 ± 21.3 for PTTG1 and 47.4 ± 17.2 for SYT1 in 10 healthy pituitary specimens by IHC. We divided 62 patients into high and low groups according to median PTTG1 or SYT1 H-scores. Higher growth hormone levels (34.5 ± 7.3 ng/mL vs. 18.4 ± 6.9 ng/mL, p < 0.05), tumor size (13.4 ± 3.6 cm^3^ vs. 6.3 ± 4.2 cm^3^, p < 0.05), and tumor recurrence (25.8% vs. 9.7%) were shown in the high-PTTG1 group compared to the low-PTTG1 group (Table 2). Higher growth hormone levels (31.4 ± 6.7 ng/mL vs. 21.5 ± 7.2 ng/mL, p < 0.05), tumor size (11.9 ± 4.1 cm^3^ vs. 7.8 ± 3.5 cm^3^, p < 0.05), and tumor recurrence (22.6% vs. 12.9%) were seen in the high-SYT1 group compared to the low-SYT1 group. There was no significant correlation between PTTG1 and SYT1 levels and age or gender. Univariate analysis showed a significant association between PTTG1 (*χ*2 = 4.510, p = 0.034) but not SYT1 (*χ*2 = 2.540, p = 0.111) expression and sparse granulation (Table 2).

### 3.5. Effects of miR-423-5pon Cell Growth, Apoptosis, Growth Hormone Release, and Migration of GH3 Cells

**Figure 4:**
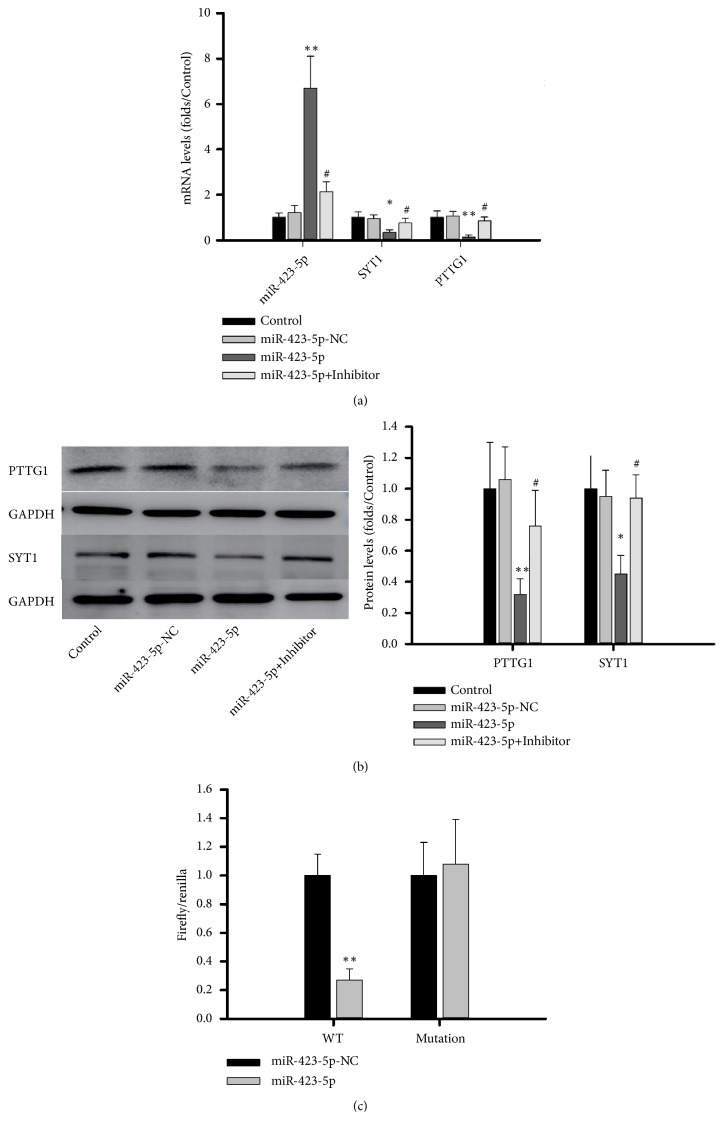
*miR-423-5p inhibited expression of PTTG1 and SYT1 in GH3 cells.* (a): mRNA levels of PTTG1 and SYT1 after 48h transfection. (b): Protein levels of PTTG1 and SYT1 after 48h transfection. (c): The activity of firefly/renilla after 48h transfection of PTTG1 wild-type or mutation plasmid. *∗* compared to control group ^*∗*^p < 0.05 ^*∗∗*^p < 0.01, #compared to miR-423-5p, p<0.05, n = 3.

To test the function of miR-423-5p in somatotroph adenoma, we transfected rat miR-423-5p mimics and a negative control miR-423-5p into the GH3 cell line. The mRNA levels of SYT1 and PTTG1 in the miR-423-5p group were 0.36 ± 0.12-fold and 0.15 ± 0.1-fold of those in the control group and restored to 0.76 ± 0.21-fold and 0.86 ± 0.16-fold in miR-423-5p+inhibitor group (Figure 4(a)). Western blots showed protein levels of 0.43 ± 0.15-fold for SYT1 and 0.23 ± 0.14-fold for PTTG1 in somatotroph adenomas compared to the control group and restored to 0.78 ± 0.3-fold and 0.94 ± 0.15-fold in miR-423-5p+inhibitor group (Figure 4(b), p< 0.05). Luciferase assay show the activity firefly/renilla in wild-type PTTG1 plasmid was 0.27±0.08-fold of that in control group and 0.93±0.31-fold in mutation PTTG1 group (Figure 4(c), p<0.05).

**Figure 5:**
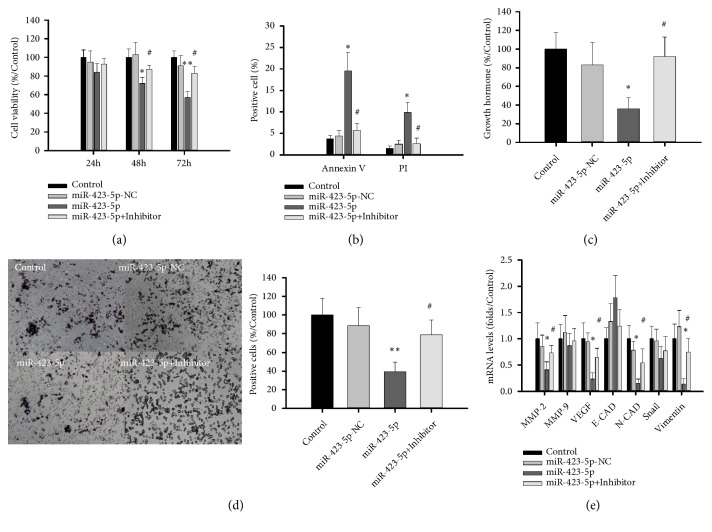
*Effect of miR-423-5pon cell growth, apoptosis, growth hormone release, and migration of GH3 cells.* (a): Viability of GH3 cells after miR-423-5ptransfection for 24h, 48h, and 72h. (b): miR-423-5p induced GH3 cell apoptosis after transfection for 24 h. (c): miR-423-5p reduced growth hormone release by GH3 cell cultures. (d): Transwell experiments showing miR-423-5p reduced transmembrane GH3 cells after transfection for 24h. (e): miR-423-5p inhibited mRNA of migration-related genes N-CAD, vimentin, VEGF, and MMP2 *∗*compared to a control group. ^*∗*^p < 0.05, ^*∗∗*^p < 0.01, #compared to miR-423-5p p<0.05, n = 3.

Cell proliferation was significantly inhibited in the miR-423-5p group compared with the miR-423-5p-NC group in MTS experiments (Figure 5(a)) (p < 0.01). Flow cytology experiments showed 19.6 ± 4.3% Annexin V-positive cells in the miR-423-5p group and 4.4 ± 1.2% in miR-423-5p-NC group (p < 0.01) and 5.7± 1.7% in miR-423-5p+inhibitor group (p<0.05). PI-positive cells were 9.8 ± 1.8% in the miR-423-5pgroup and 2.5 ± 0.9% in miR-423-5p-NC group (p < 0.01) and 2.7 ± 1.3% in miR-423-5p+inhibitor group (p<0.05). The growth hormone level in miR-423-5p group was 37.8 ± 7.3% of that in control group (p<0.01) and 92 ± 21% in miR-423-5p+inhibitor group (Figure 5(c), p<0.05). Trans-membrane assays showed that trans-membrane GH3 cells in miR-423-5p group was 39.5 ± 10.1% of that in control group (p<0.01) and 78.7 ± 15.8% in miR-423-5p+inhibitor group (Figure 5(d), p<0.05). PCR showed mRNA levels of 0.129 ± 0.11-fold for N-cadherin (N-CAD), 0.126 ± 0.08-fold for vimentin, 0.291 ± 0.15-fold for VEGF, and 0.409 ± 0.18-fold for MMP2 in miR-423-5p group compared to those of control group and restored to 0.54 ± 0.27-fold, 0.75 ± 0.25-fold, and 0.65 ± 0.17- and 0.73 ± 0.14-fold in miR-423-5p+inhibitor, respectively (Figure 5(e)).

## 4. Discussion

Somatotroph adenoma was the second functional pituitary adenomas and usually occurred with the cardiovascular, respiratory, and cerebrovascular disease, companied with a higher risk of mortality compared to normal person [21]. Surgery is the first choice of somatotroph adenomas. However, some patients were not fit for surgery or medicine, which made it difficult for the treatment of somatotroph adenomas. Exosomes have been regarded as cellular debris. Currently, exosomes are considered transporters between cells that translate messages to target cells [22]. Studies show that exosomes are related to tumorigenesis. In tumor cells, tumor-derived exosomes transport messages among cells and educated other cells, leading to tumor survival and promotion of metastasis [23]. Circulating miRNAs derived from exosomes may serve as diagnostic biomarkers by identifying specific RNA signatures of cancer cells. Exosomal miRNAs isolated from sera of OC patients have been analyzed and specific miRNA signatures discovered which may be used in the diagnosis of this cancer [24].

This study focused on exosome miRNA expression profiling in somatotroph adenomas. Between somatotroph adenomas and healthy pituitary glands, 169 miRNAs were significantly differently expressed, including hsa-miR-320a and hsa-miR-423-5p, which was proved by miRSCan PanelChip™ qPCR. These two miRNAs were identified by exosome sequencing profiling and miRSCan PCR as having lower expression level in somatotroph adenomas than in healthy pituitary adenomas. We tested the function of the two miRNAs by in vitro assay and found that miR-423-5p had important functions in GH3 cells. In vitro experiments showed that miR-423-5p inhibited cell proliferation, induced cell apoptosis, and reduced growth hormone release and migration of GH3 cells. These results demonstrated the central function of miR-423-5p in promoting tumorigenesis in somatotroph adenomas. Previous studies showed that hsa-miR-423-5p is a potential biomarker for nasopharyngeal carcinoma [25]. Transient transfection of miR-423-5p into HCC cells promotes autophagy [26]. In congestive heart failure, miR-423-5p induces apoptosis in cardiomyocytes [27]. Exosomal miR-423-5p promotes cancer growth in gastric cancer [28]. The activity of miR-423-5p on GH3 cell was nearly blocked by its inhibitor. Stiuso et al. demonstrated that cell cycle arrest, autophagy activation, and cell death were induced by overexpression of miR-423 through targeting Atg7 in hepatocellular carcinoma HuH-7 cell line [26]. Our results reached the same conclusions as previous articles about the function of miR-423-5p in promoting cancer growth in somatotroph adenomas.

Sequencing indicated that SYT1 and pituitary tumor-transforming gene (PTTG1) had interacting sequences with miR-423-5p. We found miR-423-5p inhibited expression of SYT1 and PTTG1 at the mRNA and protein levels with in vitro experiments. SYT1 had a regulatory function in membrane interactions during synaptic vesicle trafficking at the active zone of synapses [29]. In sporadic medullary thyroid cancer, proteomic analysis and Western blots show abnormal expression of SYT1 [30]. In our study, mRNA and proteomics profiling showed higher expression of SYT1 at the mRNA and protein levels in somatotroph adenomas than healthy pituitary glands. The corresponding miR-423-5p had decreased expression in somatotroph adenomas compared to healthy pituitary glands. The low levels of miR-423-5p and high levels of SYT1 matched the negative regulation of miRNA to mRNA, as reported previously. There was no database indicated that SYT1 was the target mRNA of miR-423-5p; however, there was database that proved that SYT2 was the target mRNA of miR-423-5p. SYT1 is an important paralog of SYT2 (Genecards). Paralogous genes are genes that are related by duplication from the last common ancestor of the species being compared. So we speculated that SYT1 had interacting sequence of miR-423-5p.

PTTG1 was a target mRNA of miR-423-5p and it was proved by TargetScan software. PTTG1 is highly expressed in a number of tumors to regulate tumor-related metastasis and therapeutic responses [31, 32]. In our study, mRNA and proteomics profiling showed that PTTG1 was highly expressed in somatotroph adenomas compared to healthy pituitary glands. Transient transfection of miR-423-5p into GH3 cells contributed to the lower mRNA of PTTG1 compared to miR-423-5p-NC groups. Dual luciferase reporter gene assay shown was significantly reduced in the presence of miR-423-5p in GH3 cells transfected with wild-type PTTG1 3'UTR luciferase reporter plasmid but not reduced when transfected with the mutation PTTA 3'UTR luciferase reporter plasmid. Previous studies show that PTTG1 promotes cell migration and proliferation and suppresses cell apoptosis in lung cancer [33]. High expression of PTTG1 in somatotroph adenomas may be one of the reasons for promoting cell migration and proliferation.

In conclusion, our results suggested that serum exosomal miRNAs, especially hsa-miR-423-5p, were important for GH3 cell proliferation and may have promoted tumorigenesis in somatotroph adenomas. PTTG1 were targets of miR-423-5p and acted as potential biomarkers for somatotroph adenoma therapeutic interventions through gene therapy.

## Figures and Tables

**Table 1:** Eight miRNAs differently expressed in exosome sequencing data and miRSCan PanelChipTM qPCR data.

miRNAs	PCR-FC (GH/CTL)	Sequecing-FC (GH/CTL)	True or False
hsa-miR-199a-5p	0.23	6.51	False
hsa-miR-30b-5p	0.16	2.40	False
hsa-miR-23a-3p	0.17	5.53	False
hsa-miR-223-3p	0.06	2.64	False
hsa-miR-103a-3p	0.15	2.10	False
hsa-miR-221-3p	0.19	2.97	False
*hsa-miR-320a*	*0.20*	*0.32*	*True*
*hsa-miR-423-5p*	*0.22*	*0.20*	*True*

True: miRNAs expressed both higher or lower in exosome sequencing data and miRSCan PanelChipTM qPCR data in somatotroph adenoma. False: miRNAs expressed both higher in exosome sequencing data and lower in miRSCan PanelChipTM qPCR data or expressed both lower in exosome sequencing data and higher in miRSCan PanelChipTM qPCR.

**Table 2:** Univariate and multivariate analyses for the clinicopathological correlates.

variables	Aggressiveness		univariate analysis	
	Yes(n=24)	No(n=38)	*χ* ^2^	p-value
age (years)				
≤37.6	13	18	0.272	0.602
>37.6	11	20		
gender				
Male	14	17	1.088	0.297
Female	10	21		
Tumor size(cm^3^)				
≤9.85	17	14	6.798	0.009
>9.85	7	24		
Preoperative serum				
GH level(ng/ml)				
≤26.5	16	15	4.351	0.037
>26.5	8	23		
PTTG1				
High	18	13	19.789	0.002
Low	6	25		
SYT1				
High	16	15	4.351	0.037
Low	8	23		

GH: growth hormone

High: The positive percent of PTTG1 or SYT1 is equal or more than 50%. Low: The nuclear-positive percent of PTTG1 or SYT1 is less than 50%.

## Data Availability

The Supplementary Materials data used to support the findings of this study are included within the supplementary information file.

## Supplementary Materials

Supplementary Materials Supplementary Table 1: 169 known miRNAs differently expressed between somatotroph adenomas and healthy pituitary samples; Supplementary Table 2: 169 miRNAs mapped to 6377 mRNAs; Supplementary Table 3: The target mRNAs of hsa-miR-320a and hsa-miR-423-5p; Supplementary Table 4: The target mRNAs of hsa-miR-320a and hsa-miR-423-5p by microarray analysis of somatotroph adenomas and healthy pituitary samples were used to test expression of and proteins of hsa-miR-320a and hsa-miR-423-5p. Supplementary Table 5: The target proteins of hsa-miR-320a and hsa-miR-423-5p by proteomic analysis of somatotroph adenomas and healthy pituitary samples.
